# Gene expression analysis of a *Helicobacter pylori*-infected and high-salt diet-treated mouse gastric tumor model: identification of CD177 as a novel prognostic factor in patients with gastric cancer

**DOI:** 10.1186/1471-230X-13-122

**Published:** 2013-07-30

**Authors:** Takeshi Toyoda, Tetsuya Tsukamoto, Masami Yamamoto, Hisayo Ban, Noriko Saito, Shinji Takasu, Liang Shi, Ayumi Saito, Seiji Ito, Yoshitaka Yamamura, Akiyoshi Nishikawa, Kumiko Ogawa, Takuji Tanaka, Masae Tatematsu

**Affiliations:** 1 Division of Pathology, National Institute of Health Sciences, Tokyo, Japan; 2 Division of Oncological Pathology, Aichi Cancer Center Research Institute, Nagoya, Japan; 3 Department of Pathology, Fujita Health University School of Medicine, Toyoake, Japan; 4 Faculty of Veterinary Medicine, Nippon Veterinary and Life Science University, Tokyo, Japan; 5 Chemicals Safety Department, Mitsui Chemicals Inc, Mobara, Japan; 6 Department of Pathology and Matrix Biology, Mie University Graduate School of Medicine, Tsu, Japan; 7 Department of Gastroenterological Surgery, Aichi Cancer Center Hospital, Nagoya, Japan; 8 Biological Safety Research Center, National Institute of Health Sciences, Tokyo, Japan; 9 The Tohkai Cytopathology Institute: Cancer Research and Prevention, Gifu, Japan; 10 Japan Bioassay Research Center, Hadano, Japan

**Keywords:** Cd177, Gastric cancer, *Helicobacter pylori*, Microarray, Salt

## Abstract

**Background:**

*Helicobacter pylori* (*H. pylori*) infection and excessive salt intake are known as important risk factors for stomach cancer in humans. However, interactions of these two factors with gene expression profiles during gastric carcinogenesis remain unclear. In the present study, we investigated the global gene expression associated with stomach carcinogenesis and prognosis of human gastric cancer using a mouse model.

**Methods:**

To find candidate genes involved in stomach carcinogenesis, we firstly constructed a carcinogen-induced mouse gastric tumor model combined with *H. pylori* infection and high-salt diet. C57BL/6J mice were given *N*-methyl-*N*-nitrosourea in their drinking water and sacrificed after 40 weeks. Animals of a combination group were inoculated with *H. pylori* and fed a high-salt diet. Gene expression profiles in glandular stomach of the mice were investigated by oligonucleotide microarray. Second, we examined an availability of the candidate gene as prognostic factor for human patients. Immunohistochemical analysis of CD177, one of the up-regulated genes, was performed in human advanced gastric cancer specimens to evaluate the association with prognosis.

**Results:**

The multiplicity of gastric tumor in carcinogen-treated mice was significantly increased by combination of *H. pylori* infection and high-salt diet. In the microarray analysis, 35 and 31 more than two-fold up-regulated and down-regulated genes, respectively, were detected in the *H. pylori*-infection and high-salt diet combined group compared with the other groups. Quantitative RT-PCR confirmed significant over-expression of two candidate genes including *Cd177* and *Reg3g*. On immunohistochemical analysis of CD177 in human advanced gastric cancer specimens, over-expression was evident in 33 (60.0%) of 55 cases, significantly correlating with a favorable prognosis (*P* = 0.0294). Multivariate analysis including clinicopathological factors as covariates revealed high expression of CD177 to be an independent prognostic factor for overall survival.

**Conclusions:**

These results suggest that our mouse model combined with *H. pylori* infection and high-salt diet is useful for gene expression profiling in gastric carcinogenesis, providing evidence that CD177 is a novel prognostic factor for stomach cancer. This is the first report showing a prognostic correlation between CD177 expression and solid tumor behavior.

## Background

Stomach cancer is the fourth most common cancer and second leading cause of cancer-related death worldwide [1]. *Helicobacter pylori* (*H. pylori*) is now recognized as a major risk factor for chronic gastritis and stomach cancer development [2]. In addition, environmental and host factors have also been shown to influence gastric carcinogenesis, and salt (sodium chloride, NaCl) and salty food are of particular importance, based on evidence from a number of epidemiological and experimental studies [3-6]. Thus, combined exposure to *H. pylori* infection and excessive salt intake appears to be very important for the development and progression of gastric tumors, although the detailed mechanisms, especially in terms of gene expression profiles, remain to be clarified.

High throughput microarray technology provides a powerful tool for comprehensive gene analysis, already applied to assess gene expression patterns in both human samples and animal models of gastric disorders [7-16]. Although many researchers have focused on gene expression in *H. pylori*-treated gastric cell lines [17-19], results in cell culture do not necessarily correlate with expression of specific genes in the *in vivo* microenvironment featuring host immune responses and stromal-epithelial interactions in cancers. Carcinogen-treated Mongolian gerbils have been used as a useful animal model of *H. pylori*-associated gastric carcinogenesis [20-24], and we previously reported that a synergistic interaction between *H. pylori* infection and high-salt intake accelerates chronic inflammation and tumor development in the stomachs of these animals [25,26]. Unfortunately, there is little information available for the gerbil genome, hampering genetic and molecular analysis. Therefore, attention has focused on mouse models [12,13], and establishment of a mouse model for stomach cancer featuring salt and *H. pylori* exposure is needed for investigations targeting genes involved in gastric carcinogenesis.

Previous microarray studies using rodent models did not distinguish and characterize expression profiles based on the interaction of *H. pylori* infection and salt intake. In the present study, we examined gene expression in the gastric mucosa in a *H. pylori*-infected and high-salt diet-treated mouse gastric tumor model by oligonucleotide microarray and found two candidate up-regulated genes including *Cd177* and *Reg3g*. We also investigated the expression of CD177 in human advanced gastric cancers by immunohistochemistry, and obtained evidence as a potential prognostic factor for stomach carcinogenesis.

## Methods

### Inoculation with *H. pylori*

*H. pylori* was prepared by the same method as described previously [27,28]. Briefly, *H. pylori* (Sydney strain 1) was inoculated on Brucella agar plates (Becton Dickinson, Cockeysville, MD, USA) containing 7% (v/v) heat-inactivated fetal bovine serum (FBS) and incubated at 37°C under microaerophilic conditions at high humidity for 2 days. Then, bacteria grown on the plates were introduced into Brucella broth (Becton Dickinson) supplemented with 7% (v/v) FBS and incubated under the same conditions for 24-h. After 24-h fasting, animals were intra-gastrically inoculated *H. pylori* (1.0 × 10^8^ colony-forming units). Before inoculation, the broth cultures of *H. pylori* were checked under a phase-contrast microscope for bacterial shape and mobility.

### Animals and experimental protocol

Fifty-six specific pathogen-free male, 5- or 6-week-old C57BL/6J mice (CLEA Japan, Tokyo, Japan) were used in this study. All animals were housed in plastic cages on hardwood-chip bedding in an air-conditioned biohazard room with a 12-h light/12-h dark cycle, and allowed free access to food and water throughout. The experimental design was approved by the Animal Care Committee of the Aichi Cancer Center Research Institute, and the animals were cared for in accordance with institutional guidelines as well as the Guidelines for Proper Conduct of Animal Experiments (Science Council of Japan, June 1st, 2006).

**Figure 1:**
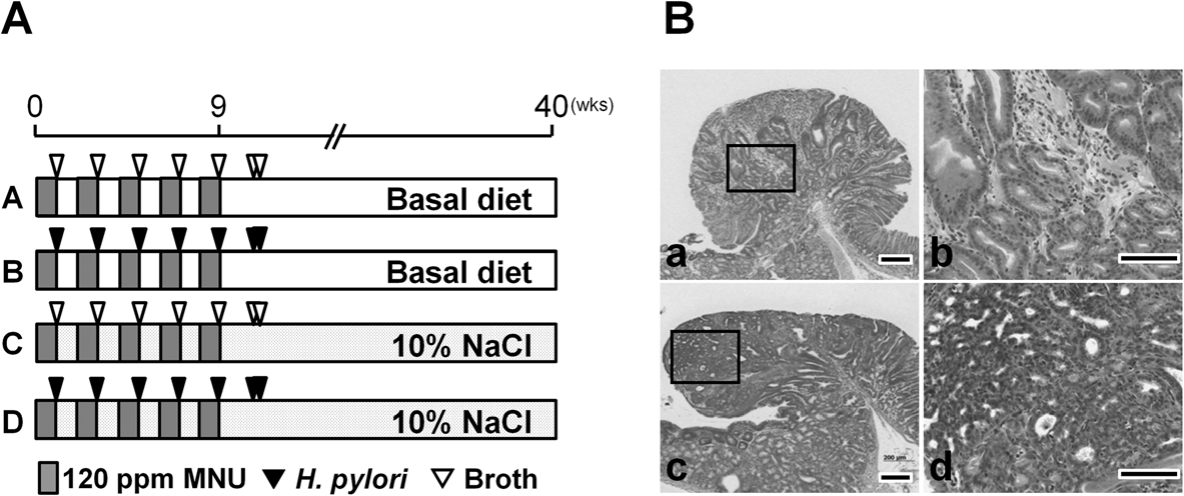
**Experimental design and histopathological findings. A**: Experimental design. Five- to six-week-old male C57BL/6J mice were inoculated with *H. pylori* SS1 strain (Groups B and D) or Brucella broth (Groups A and C). All animals were administered 120 ppm MNU in their drinking water on alternate weeks (total exposure, 5 weeks). Mice of Groups C and D were given basal diet (CE-2) containing 10% NaCl. **B**: Histopathological findings for MNU-induced mice gastric tumors. (a and b) Gastric adenoma in the pyloric region of an MNU-treated and *H. pylori*-infected mouse (Group B). (c and d) Gastric adenocarcinoma observed in Group B. Note the high cell density and cellular and structural atypia. Bar = 200 (a and c) or 100 μm (b and d).

The experimental design is illustrated in Figure 1A. The mice were divided into 4 groups (Groups A-D); 21, 5, 15, and 15 mice were assigned to A, B, C, and D groups, respectively, at the commencement of the experiment. Animals of Groups B and D were inoculated with *H. pylori* intra-gastrically on alternate weeks (total 7 times), while mice of the other groups were inoculated with Brucella broth alone. All mice were given *N*-methyl-*N*-nitrosourea (MNU, Sigma Chemical, St Louis, MO, USA) in their drinking water at the concentration of 120 ppm on alternate weeks (total exposure was 5 weeks). For this purpose MNU was freshly dissolved in distilled water three times per week. Mice of Groups C and D received CE-2 diets (basal sodium content of 0.36%; CLEA Japan) containing 10% NaCl. During the exposure period, one animal of Group B, one of Group C and six of Group D died or became moribund and they were excluded from the experiment. At 40 weeks, the remained animals were subjected to deep anesthesia and laparotomy with excision of the stomach.

### Histological evaluation

For histological examination, the stomachs were fixed in 10% neutral-buffered formalin for 24-h, sliced along the longitudinal axis into strips of equal width, and embedded in paraffin. Four-μm thick sections were prepared and stained with hematoxylin and eosin (H&E) for histological observation. Tumors were classified into adenoma and adenocarcinoma based on cellular and morphological atypia and invasive growth to submucosa as we reported previously [21].

### RNA preparation and oligonucleotide microarray analysis

Total RNA was extracted from the whole gastric mucosa including both tumor and peripheral tissue using an RNeasy Plus Mini Kit (Qiagen, Hilden, Germany) and its quality checked with a microchip electrophoresis system (i-chip SV1210; Hitachi Chemical, Tokyo, Japan). High-quality samples were selected, and pooled for each group to avoid individual difference for oligonucleotide microarray assessment (Group A, n = 3; B, n = 4; C, n = 6; D, n = 7). The CodeLink Mouse Whole Genome Bioarray (Applied Microarrays, Tempe, AZ, USA) containing 35,587 probe sets per chip was used to analyze gene expression profiles. Hybridization, processing, and scanning were performed by Filgen, Inc. (Nagoya, Japan), scan data images being analyzed using a software package (Microarray Data Analysis Tool, Filgen). Complete-linkage hierarchical clustering was also examined on the four groups using a qualified probe subset (Filgen).

### Quantitative real-time RT-PCR of expression profiles in mice stomach

Relative quantitative real-time RT-PCR was performed using a StepOne Real-Time PCR System (Applied Biosystems, Foster City, CA, USA) with the mouse-specific glyceraldehyde-3-phosphate dehydrogenase (*Gapdh*) gene as an internal control. After DNase treatment, first strand cDNAs were synthesized from total RNA using a SuperScript VILO cDNA Synthesis Kit (Invitrogen, Carlsbad, CA, USA). The PCR was accomplished basically following the manufacturer's instructions using a QuantiTect SYBR Green PCR Kit (Qiagen). The primer sequences for each gene are listed in Table 1. Specificity of the PCR reactions was confirmed using a melt curve program provided with the StepOne software and electrophoresis of the PCR samples in 3% agarose gels. The expression levels of mRNAs were normalized to the mRNA level of *Gapdh* and compared with the control mice (Group A) by the ΔΔCT method.

**Table 1:** Primer sequences for relative quantitative real-time RT-PCR

**Gene**	**Sequences**	**Product length**	**Accession no.**
*Gapdh*	5′-AACGGATTTGGCCGTATTG-3′	140	NM_008084
	5′-TTGCCGTGAGTGGAGTCATA-3′		
*Cd177*	5′-AGGGGTGCCACTCACTGTTA-3′	128	NM_026862
	5′-CCGATTGTTTTGGAGTCACC-3		
*Reg3g*	5′-GTATGGATTGGGCTCCATGA-3′	106	NM_011260
	5′-GATTCGTCTCCCAGTTGATG-3′		
*Muc13*	5′-CCTAATCCCTACGCAAACCA-3′	124	NM_010739
	5′-TCTGCCCATTTCTCCTTGTC-3′		

*Gapdh* glyceraldehyde-3-phosphate dehydrogenase, *Reg3g* regenerating islet-derived protein 3 gamma, *Muc13* mucin 13.

### Patients and tumor specimens

A total of 55 cases of primary advanced gastric cancer, surgically resected at Aichi Cancer Center Hospital (Nagoya, Japan) between 1995 and 2002, were investigated after obtaining informed consent. The study was approved by the ethics committee of Aichi Cancer Center. The patients were all male and the mean age and median follow-up period were 58.6 ± 10.2 years and 83 weeks, respectively. None had received preoperative chemotherapy or radiotherapy. Carcinomas with adjacent mucosa tissue were fixed and embedded in paraffin, and sectioned for staining with H&E. Classification of tumor staging and diagnosis of advanced cases were made according to the Japanese Classification of Gastric Carcinomas [29]. The cancers had invaded the muscularis propria (T2 for TNM classification), the subserosa (T3), or the serosa and the peritoneal cavity (T4a), sometimes involving adjacent organs (T4b).

### Immunohistochemistry using human gastric cancer tissue

We examined expression of CD177, for which a commercial primary antibody was available, in human gastric cancer tissues by immunohistochemistry. After inhibition of endogenous peroxidase activity by immersion in 3% hydrogen peroxide/methanol solution, antigen retrieval was carried out with 10 mM citrate buffer (pH 6.0) in a microwave oven for 10 min at 98°C. Then, sections were incubated with a mouse monoclonal anti-CD177 antibody (clone 4C4, diluted 1:100, Abnova, Taipei, Taiwan). Staining for CD177 was performed using a Vectastain Elite ABC Kit (Vector Laboratories, Burlingame, CA, USA) and binding visualized with 0.05% 3,3′-diaminobenzidine. The results of CD177 immunostaining in neoplastic cells were classified into four degrees; grade 0 (none, 0-10% of positive cells), grade 1 (weak, 10-30%), grade 2 (moderate, 30-60%), and grade 3 (strong, over 60%) based on proportion of stained cells, and cases showing moderate to strong staining were considered as positive.

### Statistical analysis

The Chi-square test with Bonferroni correction was used to assess incidences of gastric tumor. Quantitative values including multiplicity of tumor and relative expression of mRNA were represented as means ± SD or SE, and differences between means were statistically analyzed by ANOVA or the Kruskal-Wallis test followed by the Tukey test for multiple comparisons. Overall survival was estimated using the Kaplan-Meier method and the log-rank test for comparisons. Correlations between CD177 expression and clinicopathological factors were analyzed by ANOVA or Chi-square test. Multivariate analysis was performed to examine whether CD177 over-expression was an independent prognostic factor using the Cox proportional-hazards regression model. *P* values of < 0.05 were considered to be statistically significant.

## Results

### Incidences and multiplicities of gastric tumors

The effective number of mice and the observed incidences and multiplicities of gastric tumors are summarized in Table 2. Tumors developed in the gastric mucosa of all MNU-treated groups (Groups A-D) (Figure 1B). In high-salt diet-treated groups (Groups C and D), the incidence of gastric tumor in Group D (*H. pylori*-infected; 100%) was significantly higher than that in Group C (non-infected; 50.0%) (*P* < 0.05). In basal diet groups (Groups A and B), the incidence was also increased by *H. pylori*-infection (Group A, 61.9% and Group B, 100%), albeit without statistical significance. The multiplicities of total tumors in both *H. pylori*-infected groups (Group B, 3.3 ± 1.0 tumors/mouse and Group D, 2.6 ± 1.1) were markedly higher than those in non-infected groups (Group A, 0.9 ± 0.8 and Group C, 1.0 ± 1.2) (*P* < 0.05). The multiplicity of gastric adenocarcinoma in Group D (2.1 ± 1.4) was slightly higher than that in Group B (1.8 ± 1.0) and significantly increased over the Group C value (0.8 ± 1.0) (*P* < 0.05). In contrast, the multiplicities of adenomas in Groups A and D (0.1 ± 0.4 and 0.4 ± 0.5, respectively) were significantly lower than in Group B (1.5 ± 0.6) (*P* < 0.05 and 0.01).

**Table 2:** Incidence and multiplicity of gastric tumors in MNU-treated mice

**Group**	**Effective number**	**Treatment**	**Incidence (%)**			**Multiplicity (no. of tumor/mouse)**		
			**Adenoma**	**Carcinoma**	**Total tumor**	**Adenoma**	**Carcinoma**	**Total tumor**
A	21	MNU	3(14.3)	13(61.9)	13(61.9)	0.1 ± 0.4^a^	0.8 ± 0.7	0.9 ± 0.8
B	4	MNU + *H. pylori*	4(100)^b^	4(100)	4(100)	1.5 ± 0.6	1.8 ± 1.0	3.3 ± 1.0^c^
C	14	MNU + 10% NaCl	2(14.3)	6(42.9)	7(50.0)	0.2 ± 0.6	0.8 ± 1.0	1.0 ± 1.2
D	9	MNU + *H. pylori* + 10% NaCl	4(44.4)	8(88.8)	9(100)^d^	0.4 ± 0.5^e^	2.1 ±1.4^d^	2.6 ± 1.1^d^

Values for multiplicity are expressed as means ± SD. Incidences were generally assessed by Chi-square test, followed by pairwise analysis with Bonferroni correction. Multiplicities were generally analyzed by ANOVA, followed by the Tukey test for multiple comparison. ^a^*P* < 0.01 *vs.* Group B, ^b^*P* < 0.01 *vs.* Group A, ^c^*P* < 0.05 *vs.* Group A, ^d^*P* < 0.05 *vs.* Group C, ^e^*P* <0.05 *vs.* Group B.

### Gene expression profiling in the glandular stomachs by oligonucleotide microarray

**Figure 2:**
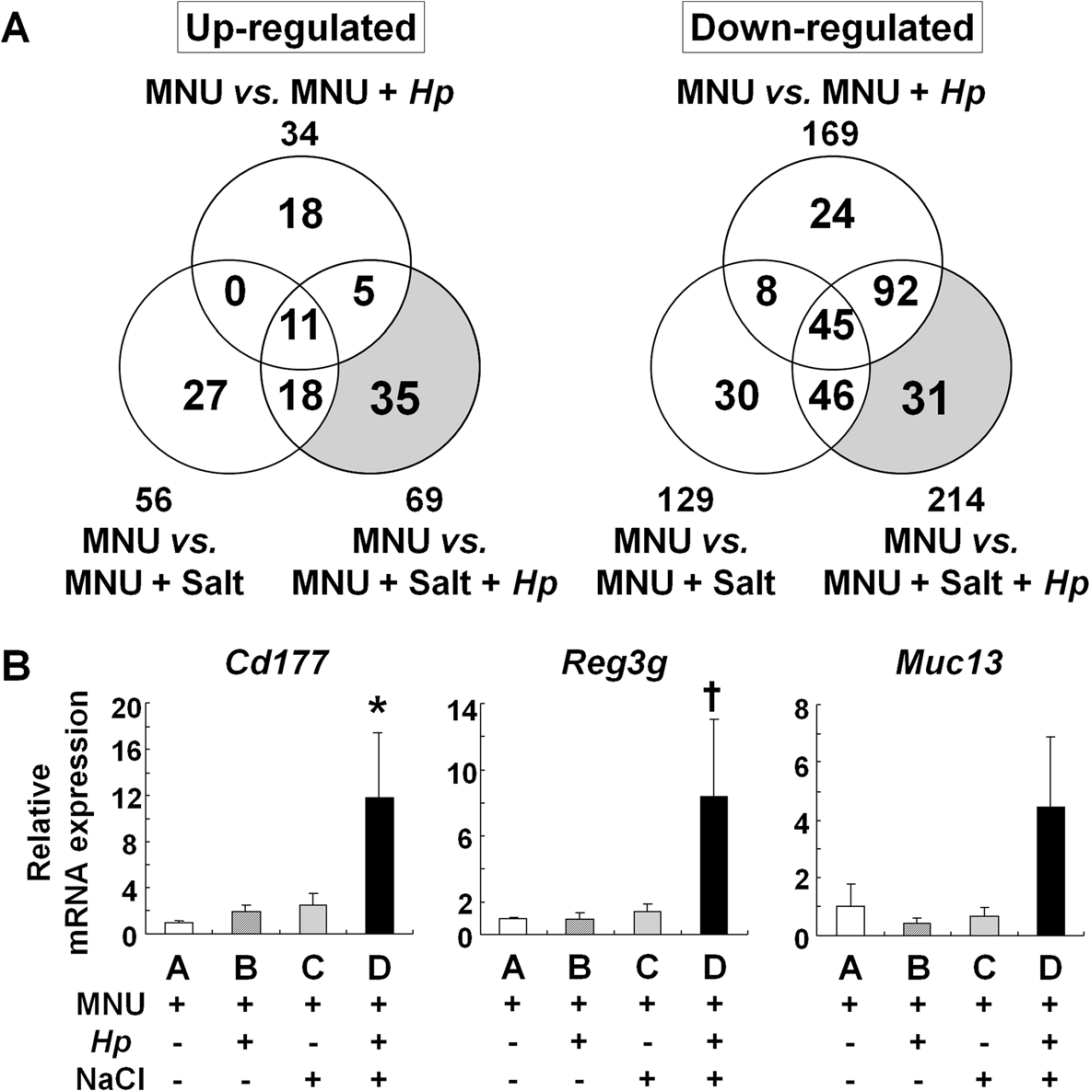
**Global gene analysis in the glandular stomach of MNU-treated mice using oligonucleotide microarray. A**: Number of genes up- or down-regulated more than two-fold in the stomach of MNU-treated mice. In Venn’s diagram, the circles indicate up- (left) or down-regulated (right) genes in the stomach of MNU-treated mice with *H. pylori* infection, high-salt diet or their combination. The shaded area represents the up- or down-regulated genes more than two-fold only by the combination. **B**: Quantitative real-time RT-PCR analysis of three selected up-regulated genes (*Cd177*, *Reg3g*, and *Muc13*) in the stomachs of MNU-treated mice. Expression levels of the genes in each sample were normalized by *Gapdh* as internal control using ΔΔCT method. Relative expression levels were represented as the X-fold change relative to Group A (fixed as 1.0). Statistical analysis was performed by the Kruskal-Wallis test for general analysis and Tukey test for multiple comparison. Bars, SE; *, *P* < 0.01 *vs.* Group A and < 0.05 *vs.* Group C; †, *P* < 0.01 *vs.* Group C.

**Figure 3:**
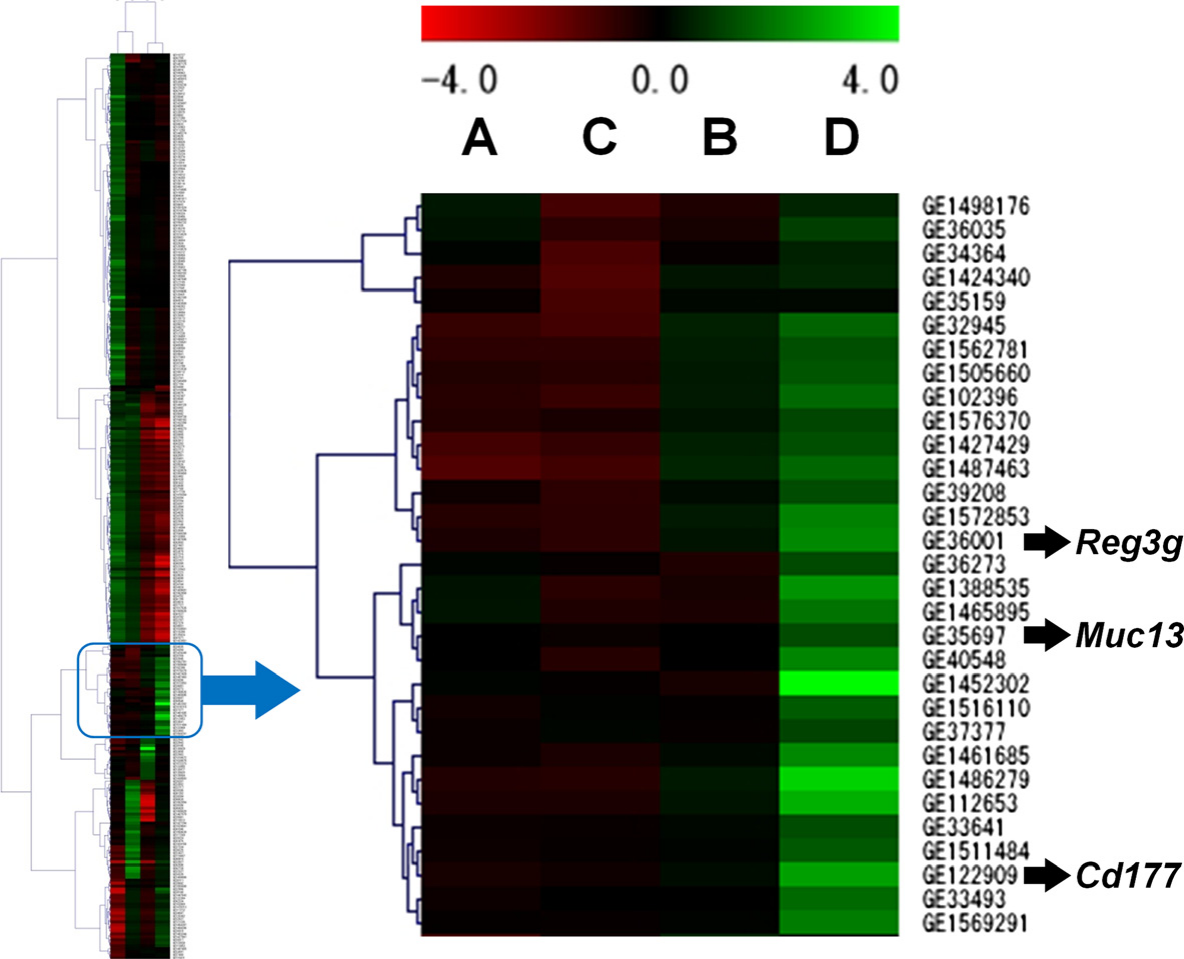
**Hierarchical clustering analysis of four experimental groups of MNU-treated mice.** Expression data from 303 qualified probes (left). The four experimental groups were classified into two clusters (Groups A/C) and (Groups B/D) based on similarities in expression patterns. Each row represents a probe and each column represents a experimental group (Groups A-D). As shown in the color bar, green indicates up-regulation; red indicates down-regulation; and black indicates no change. Thirty-one probes constituted a cluster of probes up-regulated only in Group D (right).

With oligonucleotide microarrays, compared with the non-infected and basal diet-treated group (Group A), 34 genes were up-regulated and 169 were down-regulated more than two-fold in *H. pylori*-infected mice (Group B), 56 up-regulated and 129 down-regulated in high-salt diet-treated mice (Group C), and 69 up-regulated and 214 down-regulated in the combined group (Group D) (Figure 2A). Taken together, as shown in Table 3, we found that 35 genes were up-regulated and 31 genes were down-regulated more than two-fold only by the combination of *H. pylori* infection and high-salt diet. In addition, hierarchical clustering analysis was performed on the four groups with a total of 303 qualified probes using the complete-linkage clustering algorithm (Figure 3). Thirty-one probes including *Cd177*, *Reg3g* and *Muc13* were confirmed to be within a cluster of probes up-regulated only in Group D. Subsequent analysis in the present study was focused on these genes, because it was considered that the genes in which expression was altered only in the combined group might be associated with gastric carcinogenesis and progression in humans.

**Table 3:** **Regulated genes by combination of *H. pylori *infection and high-salt diet in mouse gastric mucosa**

**Accession no.**	**Symbol**	**Genes/proteins**	**Fold changes**
*Up-regulated genes*			
XM_357640	Igk-V8	Immunoglobulin kappa chain variable 8 (V8)	14.4
XM_001472541	Ighg	Immunoglobulin heavy chain (gamma polypeptide)	9.2
NM_026862	Cd177	CD177 antigen	7.3
NM_011260	Reg3g	Regenerating islet-derived 3 gamma	6.1
NM_023137	Ubd	Ubiquitin D	4.3
XM_144817	Igk-V34	Immunoglobulin kappa chain variable 34 (V34)	4.1
NM_007675	Ceacam10	Carcinoembryonic antigen-related cell adhesion molecule 10	3.7
NM_183322	Khdc1a	KH domain containing 1A	3.3
NM_011475	Sprr2i	Small proline-rich protein 2I	3.2
NM_175165	Tprg	Transformation related protein 63 regulated	3.2
NM_175406	Atp6v0d2	ATPase, H+ transporting, lysosomal V0 subunit D2	3.0
NM_009703	Araf	v-raf murine sarcoma 3611 viral oncogene homolog	2.6
NM_026822	Lce1b	Late cornified envelope 1B	2.5
NM_016958	Krt14	Keratin 14	2.5
NM_212487	Krt78	Keratin 78	2.4
NM_009807	Casp1	Caspase 1	2.4
NM_146037	Kcnk13	Potassium channel, subfamily K, member 13	2.4
NM_019450	Il1f6	Interleukin 1 family, member 6	2.3
NM_008827	Pgf	Placental growth factor	2.3
XM_893506	Klk12	Kallikrein related-peptidase 12	2.3
NM_016887	Cldn7	Claudin 7	2.3
NM_029360	Tm4sf5	Transmembrane 4 superfamily member 5	2.2
NM_172301	Ccnb1	Cyclin B1	2.2
NM_010739	Muc13	Mucin 13, epithelial transmembrane	2.2
NM_011165	Prl4a1	Prolactin family 4, subfamily a, member 1	2.2
NM_010162	Ext1	Exostoses (multiple) 1	2.2
NM_011704	Vnn1	Vanin 1	2.1
NM_011082	Pigr	Polymeric immunoglobulin receptor	2.1
NM_007769	Dmbt1	Deleted in malignant brain tumors 1	2.1
NM_022984	Retn	Resistin	2.1
NM_173037	Tmco 7	Transmembrane and coiled-coil domain 7	2.1
NM_009100	Rptn	Repetin	2.1
NM_007630	Ccnb2	Cyclin B2	2.1
NM_001081060	Slc9a3	Solute carrier family 9 (sodium/hydrogen exchanger), member 3	2.0
NM_146588	Olfr1030	Olfactory receptor 1030	2.0
*Down-regulated genes*			
NM_008753	Oaz1	Ornithine decarboxylase antizyme 1	0.31
NM_027126	Hfe2	Hemochromatosis type 2 (juvenile) (human homolog)	0.33
NM_053206	Magee2	Melanoma antigen, family E, 2	0.33
NM_010924	Nnmt	Nicotinamide N-methyltransferase	0.41
NM_026260	Tctn3	Tectonic family member 3	0.41
NM_181039	Lphn1	Latrophilin 1	0.43
NM_008312	Htr2c	5-hydroxytryptamine (serotonin) receptor 2C	0.43
NM_146667	Olfr740	Olfactory receptor 740	0.44
NM_007550	Blm	Bloom syndrome homolog (human)	0.44
NM_011243	Rarb	Retinoic acid receptor, beta	0.44
NM_184052	Igf1	Insulin-like growth factor 1	0.45
NM_013893	Reg3d	Regenerating islet-derived 3 delta	0.46
NM_008645	Mug1	Murinoglobulin 1	0.46
NM_029550	Keg1	Kidney expressed gene 1	0.46
NM_019388	Cd86	CD86 antigen	0.46
NM_011316	Saa4	Serum amyloid A 4	0.47
NM_007811	Cyp26a1	Cytochrome P450, family 26, subfamily a, polypeptide 1	0.47
NM_011538	Tbx6	T-box 6	0.48
NM_011086	Pip5k3	Phosphatidylinositol-3-phospate/phosphatidylinositol 5-kinase, type III	0.48
NM_133723	Asph	Aspartate-beta-hydroxylase	0.48
NM_001081390	Palld	Palladin, cytoskeletal associated protein	0.48
NM_007858	Diap1	Diaphanous homolog 1 (Drosophila)	0.48
NM_053271	Rims2	Regulating synaptic membrane exocytosis 2	0.48
NM_153163	Cadps2	Ca2+-dependent activator protein for secretion 2	0.49
NM_007541	Bglap1	Bone gamma carboxyglutamate protein 1	0.49
NM_031871	Ghdc	GH3 domain containing	0.49
NM_025545	Aptx	Aprataxin	0.49
NM_177322	Agtr1a	Angiotensin II receptor, type 1a	0.49
NM_026872	Ubap2	Ubiquitin-associated protein 2	0.49
NM_028045	Erv3	Endogenous retroviral sequence 3	0.49
NM_011641	Trp63	Transformation related protein 63	0.49

The entire results of this microarray analysis have been submitted and are readily retrievable from the public database NCBI Gene Expression Omnibus (GEO) with the accession number GSE29444 (sample number: GSM728857-60).

### Quantitative real-time RT-PCR analysis of gene expression profiles in MNU-treated mouse stomachs

Relative quantitative real-time RT-PCR analysis of three selected up-regulated genes (*Cd177*, *Reg3g*, and *Muc13*) in *H. pylori*-infected and high-salt diet-treated mice confirmed increased expression of *Cd177* and *Reg3g*, as shown in Figure 2B, with significant differences. Although expression level of *Muc13* in Group D was higher than all other groups, there was no statistical significance among them (*P* = 0.0712 *vs.* Group C).

### Immunohistochemical expression of CD177 in human advanced gastric cancers and correlation with clinicopathological factors

**Figure 4:**
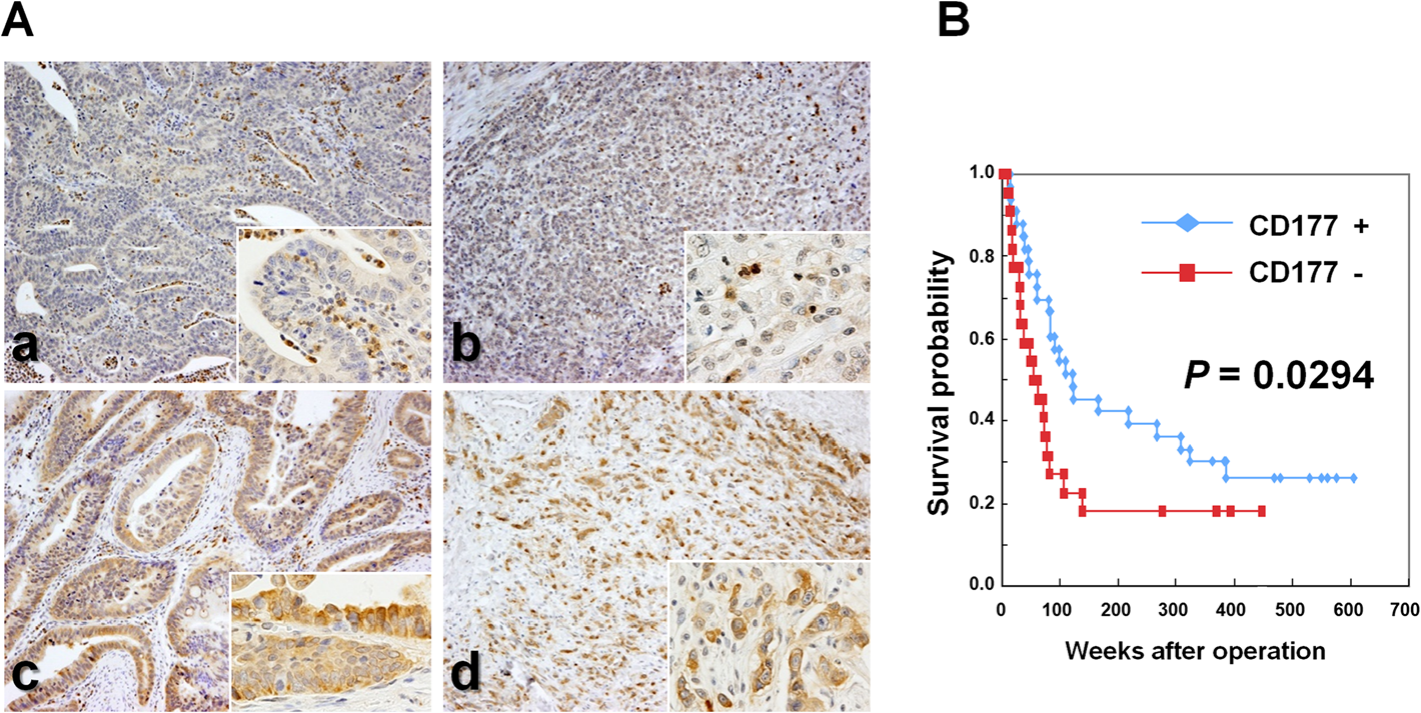
**Immunohistochemistry for CD177 in human advanced gastric cancer and correlation with overall survival rate. A**: Immunohistochemical analysis of CD177 expression in human gastric cancer tissue. (a and b) Negative staining (none to weak) for CD177 in a gastric adenocarcinoma. CD177 expression is present only in infiltrating neutrophils while neoplastic cells of well-differentiated (a) or poorly-differentiated (b) carcinoma are negative. Original magnification, ×100 (inset, ×400). (c and d) Note positive (moderate to strong) expression for CD177 in well-differentiated (c) or poorly-differentiated (d) gastric cancer cells. Original magnification, ×100 (inset, ×400). **B**: Comparison of Kaplan-Meier cumulative survival curves for CD177 negative and positive gastric cancer cases.

On immunohistochemical analysis of human gastric cancer tissues, CD177 was observed not only in the membranes and cytoplasms of infiltrated neutrophils, but also in gastric cancer cells of both well- and poorly-differentiated adenocarcinomas (Figure 4A). Cancer cells of signet-ring cell type (2 cases) were negative for CD177. Among 55 gastric cancer cases, moderate to strong expression of CD177 was observed in 33 (60.0%) (Table 4).

**Table 4:** CD177 expression in gastric carcinomas and its correlation with clinicopathological factors

	**Case no.**	**CD177 Over-expression**				***P *value‡**
		**Positive**		**Negative**		
		**Strong**	**Moderate**	**Weak**	**None**	
Gastric adenocarcinomas	55	18	15	17	5	
Age						
Years (means ± SD)		55.3 ± 10.4	60.2 ± 8.13	59.8 ± 11.0	60.4 ± 13.0	0.5039
Histological classification						
Well/moderately-differentiated type*	21	6	9	4	2	0.1904
Poorly-differentiated/Signet-ring cell type**	34	12	6	13	3	
Depth of invasion†						
T1-3	27	5	10	10	2	0.2011
T4	26	11	5	7	3	
Lymph node metastasis						
N0	6	1	2	2	1	0.7869
N1-3	49	17	13	15	4	

* Lauren’s intestinal type, ** Lauren’s diffuse type, † Case number was reduced to fifty-three because the depth of invasion was not classified in two cases, ‡ ANOVA and Chi-square test were performed for age and other factors, respectively.

The follow-up period of the patients ranged from 9 to 606 weeks (median = 83 weeks). Five-year survival rates for CD177-positive and negative were 39.4% and 18.2%, respectively. From the Kaplan-Meier survival curve analysis, CD177-positive expression was associated with better overall survival (*P* = 0.0294, log-rank test) (Figure 4B). There was no statistically significant correlation of CD177 expression with age, histological classification, depth of invasion, and lymph node metastasis (Table 4).

### Multivariate analysis for overall survival of human gastric cancer cases

Using the Cox proportional hazards model, multivariate analysis of clinicopathological variables, including the patient age, tumor histological classification, invasion depth, lymph node metastasis, and CD177 expression (Table 5), revealed the last to be an independent factor for overall survival (*P* = 0.0323). Patient age and low differentiation of adenocarcinoma were also associated with poor overall survival (*P* = 0.0439 and 0.0017, respectively). Tumor invasion depth and lymph node metastasis were not independent factors of gastric cancer cases in the present study (*P* > 0.05).

**Table 5:** Multivariate analysis of prognostic factos in patients with gastric cancer using Cox proportional hazard model

**Factors**	**Hazard ratio**	**95% CI**	***P *value**
CD177 expression (negative)	2.07	1.063-4.021	0.0323
Age (year)	1.04	1.001-1.071	0.0439
Histological type (poorly-differentiated)	4.06	1.695-9.742	0.0017
Depth of invasion (high grade)	1.64	0.790-3.410	0.1838
Lymph node metastasis (positive)	3.40	0.773-14.92	0.1055

## Discussion

In the present study, we demonstrated that the mouse model combined with *H. pylori* infection and high-salt diet is a useful tool to investigate the detailed mechanisms both of development and progression of gastric neoplasms. A number of rodent models of gastric cancer have been developed under various conditions, including *H. pylori* or *H. felis* infection, exposure to chemical carcinogens, and genetic modification [21,30]. Since *H. pylori* is known as a most closely-associated risk factor in man, animal models with infection of the bacterium, such as that utilizing Mongolian gerbils, are considered to be particularly important to mimic the background of human gastric carcinogenesis. On the other hand, there is a consensus that gastric cancer is a multifactorial disease [31]. Epidemiological studies and animal experiments have demonstrated that development of stomach cancer is also associated with many other factors including salt intake, alcohol drinking and cigarette, containing a wide variety of chemical carcinogen. In the present study, we attempted to mimic the gastric environment of human high-risk group exposed to combination of *H. pylori* infection, salt intake, and carcinogen.

As might be expected, there are both advantages and disadvantages of *Helicobacter*-infected mouse models. Instability of *cag* pathogenicity islands (PAI), a particularly important virulence factor of *H. pylori*, has been reported in the mouse model using SS1 strain [32]. Multiplicity of gastric tumors is difficult to examine in the gerbil model, because almost all of the stomach tumors in gerbils show invasive growth into the lamina propria or muscle layer. In the present study, our results demonstrated that *H. pylori* infection increased not only incidence but also multiplicity of gastric tumors in MNU-treated mice. Thus, the mouse model presented here has advantages in respect to investigate the multiplicity and tissue sampling for gene expression analysis.

In this study, we focused on the genes in which the expression was regulated only in *H. pylori*-infection and high-salt diet combined mice, which are expected to reflect the background of human high-risk group, to explore examples which might be associated with tumor progression. The two up-regulated genes selected, *Cd177* and *Reg3g* could be confirmed to exhibit significant over-expression by relative quantitative RT-PCR. Expression level of *Muc13* showed a tendency for increase with combination of *H. pylori* and salt, although this was not statistically significant. *Muc13* is a recently identified gene encoding transmembrane mucin that is expressed in the stomach to large intestine [33]. Shimamura et al. have reported that overexpression of *Muc13* is associated with differentiation towards the intestinal (differentiated) type of human gastric cancer [34]. In addition, the combined expression of MUC13 with other metaplasia biomarkers is shown to be a prognostic indicator in several types of gastric cancer [35]. In the present study, all gastric tumors observed in MNU-treated mice were histologically of differentiated type. The REG protein family is also known to be associated with gastric cancer development and *Reg1α* and *Reg4* have been suggested as prognostic markers for advanced stomach cancers in man [36]. The present results indicate the possibility that *Reg3g* is also involved with progression of stomach tumor.

Immunohistochemical analysis of CD177 in advanced gastric cancer specimens showed expression to be significantly correlated with a good prognosis and survival rate after surgery. Importantly, multivariate analysis with clinicopathological factors as covariates further revealed high expression to be an independent prognostic factor for overall survival, as along with patient’s age and histological classification. To our knowledge, the present study is the first to provide evidence that high expression of CD177 is associated with favorable prognosis in advanced gastric cancer.

CD177 is a member of the leukocyte antigen 6 (Ly-6) gene superfamily, encoding two neutrophil-associated proteins, NB1 and PRV-1 [37,38]. The NB1 glycoprotein is typically expressed on a subpopulation of neutrophils, located at plasma membranes and secondary granules. Recent studies have demonstrated that CD177 is over-expressed in neutrophils from 95% of patients with polycythemia vera and in half of patients with essential thrombocythemia [37]. Gonda et al. have reported a microarray analysis that *Cd177* expression in whole gastric tissue of *H. felis*-infected mice with mucosal dysplasia is reduced by folic acid supplementation [39]. Because they compared stage-matched groups to detect up- or down-regulated genes only by treatment of folic acid, it is unclear if *Cd177* expression is associated with gastritis or dysplasia. In our microarray results, there were no significant differences in expression of *Ela2*, which is a neutrophil-specific gene [40], and histological degrees of neutrophil infiltration were almost same among *H. pylori*-infected groups (data not shown). Therefore, the up-regulation of *Cd177* observed in this study was considered to be caused not by increased infiltration of neutrophils into the gastric mucosa but by a change of gene expression in tumor cells. NB1 is similar in structure to urokinase-type plasminogen activator receptor (uPAR), which is known to be associated with cell adhesion and migration [37]. Thus, there is a possibility that CD177 also acts as a regulator of adhesion and migration of neoplastic cells in gastric tumor. Further studies are needed to clarify the association between CD177 expression in gastric epithelial cells and tumor progression.

## Conclusions

We demonstrated that the mouse model combined with *H. pylori* infection and high-salt diet is suitable for investigation of global gene expression associated with gastric tumor development and progression. Furthermore, our results suggest that CD177 expression might be associated with a favorable prognosis of gastric adenocarcinomas in man.

## Abbreviations

FBS: Fetal bovine serum; Gapdh: Glyceraldehyde-3-phosphate dehydrogenase; H&E: Hematoxylin and eosin; H. pylori: *Helicobacter pylori*; Ly-6: Leukocyte antigen 6; MNU: *N*-methyl-*N*-nitrosourea; Muc13: Mucin 13; PAI: Pathogenicity islands; Reg3g: Regenerating islet-derived 3 gamma; RT-PCR: Reverse transcription-polymerase chain reaction; uPAR: Urokinase-type plasminogen activator receptor.

## Competing interests

The authors declare that they have no competing interests.

## Authors’ contributions

TTo and TTs designed the study under the supervision of AN, KO, TTa and MT. MY, HB, NS, ST, LS and AS participated in the animal handling and procedures. Clinical sample collection and suggestions were provided by SI and YY. Sample analysis and evaluation were performed by TTo, TTs and MY. All authors read and approved the final manuscript.

## Pre-publication history

The pre-publication history for this paper can be accessed here:

http://www.biomedcentral.com/1471-230X/13/122/prepub
